# Neutrophil-to-lymphocyte ratio, monocyte-to-lymphocyte ratio, platelet-to-lymphocyte ratio associated with 28-day all-cause mortality in septic patients with coronary artery disease: a retrospective analysis of MIMIC-IV database

**DOI:** 10.1186/s12879-024-09516-5

**Published:** 2024-07-29

**Authors:** Xicong Li, Yubiao Chen, Qi Yuan, Hongya Zhou, Lifei Lu, Ruiwei Guo

**Affiliations:** 1 Department of Cardiology, the 920th Hospital, Kunming Medical University, Kunming, 650032 Yunnan China; 2 State Key Laboratory of Respiratory Disease, National Center for Respiratory Medicine, National Clinical Research Center for Respiratory Disease, Guangzhou Institute of Respiratory Health, The First Affiliated Hospital of Guangzhou Medical University, 151 Yanjiang Road, Guangzhou, Guangdong China; 3 Department of Cardiology, 920th Hospital of Joint Logistics Support Force, PLA, Kunming, 650032 Yunnan China; 4 Guangzhou National Laboratory, Guangzhou, China

**Keywords:** Biomarker, Coronary artery disease, Sepsis, Sequential organ failure assessment scores

## Abstract

**Background:**

High Neutrophil-to-Lymphocyte Ratio (NLR), Monocyte-to-Lymphocyte Ratio (MLR), Platelet-to-Lymphocyte Ratio (PLR) were associated with worse prognosis of patients with sepsis. In-hospital mortality has been reported to be higher in patients with coronary artery disease (CAD) and sepsis than those with sepsis alone. However, the relationship between NLR, MLR, PLR and mortality in septic patients with coronary artery disease (CAD) remains unclear. The study aimed to explore the association between NLR, MLR, PLR and 28-day all-cause mortality in septic patients with CAD.

**Methods:**

We performed an observational cohort study of septic patients with CAD from the Medical Information Mart for Intensive Care (MIMIC)-IV database between 2008 and 2019. The patients were categorized by three group (Q1: low levels, Q2: medium levels, Q3: high levels) based on tertiles of NLR, MLR, and PLR. The associations between NLR, MLR, PLR and 28-day all-cause mortality were examined using the Cox proportional hazards model. Subsequently, we applied receiver operating characteristic (ROC) analysis for predicting 28-day mortality in septic patients with CAD by combining NLR, MLR and PLR with the modified sequential organ failure assessment (mSOFA) scores.

**Results:**

Overall 1,175 septic patients with CAD were included in the study. Observed all-cause mortality rates in 28 days were 27.1%. Multivariate Cox proportional hazards regression analysis results showed that 28-day all-cause mortality of septic patients with CAD was significantly related to rising NLR levels (adjusted hazard ratio [aHR]: 1.02; 95% confidence interval [CI]: 1.01–1.02; *P* < 0.001), MLR levels (aHR: 1.29; 95%CI: 1.18–1.41; *P* < 0.001), and PLR levels (aHR: 1.0007; 95%CI: 1.0004–1.0011; *P* < 0.001). Meanwhile, the higher levels (Q3) group of NLR, MLR, and PLR also had a higher risk of 28-day all-cause mortality than the lower (Q1) group. The area under the ROC curve of NLR, MLR, PLR, and mSOFA score were 0.630 (95%CI 0.595–0.665), 0.611 (95%CI 0.576–0.646), 0.601 (95%CI 0.567–0.636) and 0.718 (95%CI 0.689–0.748), respectively. Combining NLR, MLR, and PLR with mSOFA scores may improve ability of predicting 28-day mortality (AUC: 0.737, 95%CI 0.709–0.766).

**Conclusion:**

Higher levels of NLR, MLR and PLR were associated with 28-day all-cause mortality in septic patients with CAD. Further investigation will be needed to improve understanding of the pathophysiology of this relationship.

## Introduction

Sepsis is the leading cause of mortality in intensive care units (ICUs), responsible for approximately 30–50% of deaths [1, 2]. Despite the pathophysiology of sepsis remains unclear, organ damage induced by inflammation is well recognized [3, 4]. The cardiovascular system is especially vulnerable to damage during sepsis, and decreased cardiac function has been linked to heightened inflammation, oxidative stress, apoptosis, and restricted autophagy in sepsis [5]. Moreover, heightened endothelial injury has been observed in septic patients with coronary artery disease (CAD) compared to those without CAD [3]. Additionally, a large-scale, real-world cohort study involving over 2.6 million individuals revealed that acute myocardial infarction (AMI) contributed to approximately 4.5% of deaths and was linked to higher mortality rates in patients with sepsis [6]. The recent emphasis of ICU management has been on septic patients with CAD, but the early identification of the poor prognosis associated with these specially subgroup disease remains a challenge for ICU clinicians [7].

Clinical biomarkers derived from laboratory test results have recently played a pivotal role in objectively evaluating disease severity and forecasting clinical prognosis in patients [8–10]. Blood routine examination and biochemistry have garnered considerable attention due to their inclusion as routine test parameters. Neutrophil-to-lymphocyte ratio (NLR), monocyte-to-lymphocyte ratio (MLR), and platelet-to-lymphocyte ratio (PLR) are commonly utilized inflammatory biomarkers easily obtained from blood routine and biochemistry to evaluate inflammatory activity [11–15]. Inflammatory biomarkers have been demonstrated to predict mortality risk in patients with sepsis [16]. For instance, NLR and PLR have been identified as predictors of 90-day mortality in patients with sepsis [17]. Some meta-analysis showed higher NLR and PLR values may indicate unfavorable prognosis in patients with sepsis [18–20]. In patients with CAD, several studies have indicated a correlation between these markers and the severity, prognosis, and presence of CAD [11, 21]; Elevated NLR has been linked to mortality in patients with AMI [22]. Post-AMI patients who do not undergo percutaneous coronary intervention (PCI) may demonstrate an increased inflammatory response and elevated NLR and PLR levels, which are linked to decreased left ventricular thrombus (LVT) resolution despite anticoagulant therapy [23]. Nevertheless, the associations between NLR, MLR, PLR and mortality in septic patients with CAD remains unclear. Additionally, the modified Sequential Organ Failure Assessment (mSOFA) score, as a rapid and comprehensive assessment of sepsis-related organ failure, has been linked to mortality in septic patients [24]. Nonetheless, the predictive value of NLR, MLR, and PLR in combination with mSOFA scores for 28-day mortality in septic patients with CAD is still uncertain.

Hence, the main objectives of this study were : (1) to examine the relationship between NLR, MLR, PLR and 28-day all-cause mortality in septic patients with CAD; and (2) to employ receiver-operator characteristic (ROC) curves to assess the predictive ability of combining NLR, MLR and PLR with mSOFA scores in predicting the 28-day all-cause mortality in septic patients with CAD.

## Methods

### Study design and population

The study data were based on the Medical Information Mart for Intensive Care (MIMIC-IV 2.2 version) database provided information on 315,460 inpatients of Beth Israel Deaconess Medical Center from 2008 to 2019 for the American cohort [25]. Patients were included meeting the criteria as following: (1) Patients were diagnosed as sepsis within 12 h before or 24 h after ICU admission [26]. (2) combined with CAD; (3) Blood routine and biochemistry measured at ICU admission within 6 h before or 24 h after ICU admission. While patients meeting the following criteria were excluded: (1) insufficient or missing important demographic information and laboratory results (Body Mass Index [BMI], NLR, MLR and PLR); (2) be diagnosed as hematological disease.

To safeguard patient privacy, all personally identifiable information has been anonymized. As all patient records in the MIMIC-IV database were thoroughly de-identified, the need for individual patient consent was deemed unnecessary by the institutional review board of the Beth Israel Deaconess Medical Center, and the study was conducted out in conformity with the Declaration of Helsinki.

### Data collection

To access the database, we had gone through a “CITI Data or Specimens Only Research” training course on the National Institutes of Health website, the author was approved to extract data from this database for research purposes (Certificate No: 40,416,369). We extracted variables including demographics, comorbidities, laboratory results, laboratory indices, and discharge status from MIMIC-IV database. Diabetes was defined based on ICD-9 codes 250.0x-250.9x and ICD-10 codes E10-E14. The code for data query and extraction was available from the Repository (website: https://github.com/MIT-LCP/mimic-code). Sepsis was defined according to The Third International Consensus Definitions for Sepsis and Septic Shock [27]. In brief, patients with documented or suspected infection and an acute change in total SOFA score of ≥ 2 points were considered to have sepsis [28]. The code used to extract sepsis patients who meet the sepsis 3.0 standard can be found at https://github.com/alistairewj/sepsis3-mimic. CAD was defined according to the codes 410–411 (ICD-9) and I20-I21 (ICD-10) (https://icd.who.int/browse10/2019/en) [29–32] from disease history and medical record diagnosis, including previous myocardial infarction, or acute coronary syndrome etc. Only the initial admission was taken into account if a patient had multiple admissions.

### Definition of NLR, MLR, and PLR

Laboratory test parameters included neutrophil count, monocyte count, lymphocyte count and platelet count. We applied for the average value of laboratory tests in the range from 6 h before admission to 24 h after admission to ICU. Definitions of NLR, MLR, and PLR were as following [11]:

NLR—neutrophil/lymphocyte ratio;

PLR—platelet/lymphocyte ratio;

MLR—monocyte/lymphocyte ratio;

The patients were categorized by three group (Q1: low levels, Q2: medium levels, Q3: high levels) based on triplicate of NLR (Q1 < 5.86, 5.86 ≤ Q2 < 12.58, and Q3 ≥ 12.58), MLR (Q1 < 0.32, 0.32 ≤ Q2 < 0.70, and Q3 ≥ 0.70), and PLR (Q1 < 1.90, 1.90 ≤ Q2 < 90.21, and Q3 ≥ 90.21). The end points of the study included all-cause mortality at 7, 14, 21 and 28 days.

### Statistical analysis

SPSS statistical version 26.0 (IBM Corp. Armonk, NY, USA) and R software (version 4.2.1) was performed for the statistical analysis. Continuous variables having a normal distribution were presented as the mean standard deviation (SD), whereas those without a normal distribution were described as the median [interquartile range (IQR)]. Student’s t-test, Wilcoxon rank sum test, and Chi-square test were used to evaluate baseline characteristics and inflammatory biomarkers, while the Kaplan-Meier technique with the log-rank test was used to assess 28-day all-cause mortality. The relationship between NLR, MLR, PLR and 28-day mortality in septic patients with CAD was examined using the Cox proportional hazards model, adjusting for age, gender, BMI, race, cardiovascular disease, chronic pulmonary disease, liver disease, malignancy, dementia, mechanical ventilation. We applied receiver operating characteristic (ROC) analysis for predicting 28-day all-cause mortality in septic patients with CAD by combining NLR, MLR and PLR with mSOFA scores. The Delong’s test was performed to compare different ROC curves. *P* < 0.05 was considered statistically significant.

## Results

### Patient characteristics

**Fig. 1:**
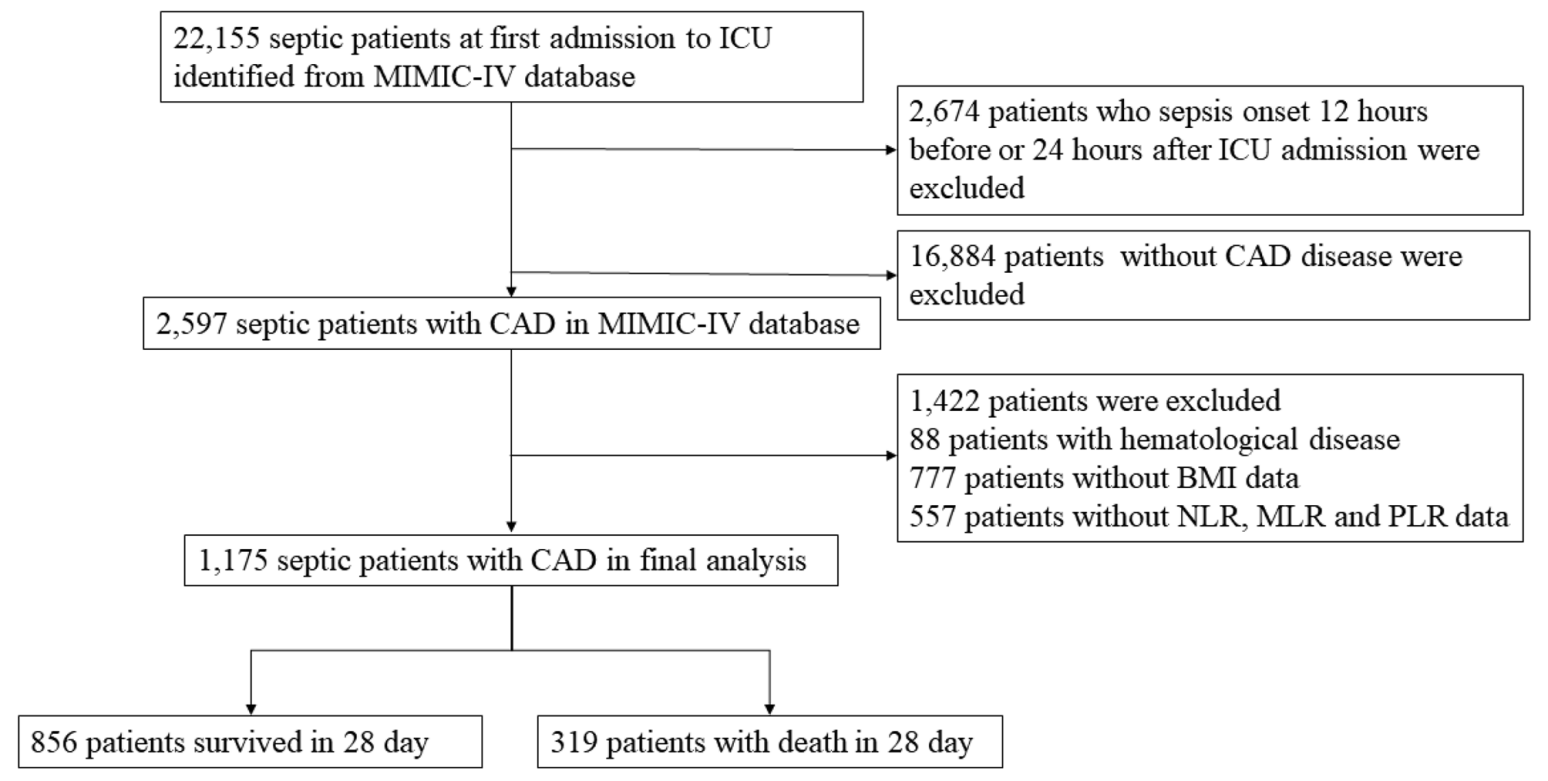
The flow chart of inclusion and exclusion

The flow chart of inclusion and exclusion are shown in Fig. 1. This study included 22,155 patients with sepsis from the MIMIC-IV database. 2,674 patients who were identified as sepsis 12 h before or 24 h after ICU admission were excluded; 16,884 patients without CAD disease were excluded. Then 1,422 patients due to hematological disease and the lack of important demographic information and laboratory results were excluded. Finally, a total of 1,175 septic patients with CAD were included in this study. The mean age of overall patients was 71.46 (62.32–79.65) years, and 63.7% of patients were male. Among the 1,175 patients, 856 (72.9%) survived 28 days, while 319 (27.1%) died within 28 days.

Table 1 shows the baseline characteristics of the survival and non-survival groups. Compared with the survival group, the non-survival group was older (75.39 [66.28, 83.76] vs. 69.99 [61.66, 78.53], *P* < 0.001), and had a lower BMI (27.09 [23.54, 32.26] vs. 28.37 [24.81, 32.67], *P* < 0.001), higher proportion of liver disease (18.2% vs. 9.5%, *P* < 0.001), malignancy (11.9% vs. 6.1%, *P* = 0.001), higher charlson comorbidity Index (CCI) (8 [6–9] vs. 7 [5–8], *P* < 0.001) and mSOFA scores (6 [4–8] vs. 4 [2–6], *P* < 0.001). No differences between race and other comorbidities (cardiovascular disease, chronic pulmonary disease, renal disease, diabetes, dementia, and acquired Immunodeficiency Syndrome [AIDS]) were observed in both groups (*P* > 0.05).

**Table 1:** Baseline characteristics

Variable	Total (*n* = 1175)	Survival (*n* = 856)	Non-survival (*n* = 319)	*p*
Age (y)	71.46 (62.32, 79.65)	69.99 (61.66, 78.53)	75.39 (66.28, 83.76)	**< 0.001**
Gender (%)				**0.031**
Female	426 (36.3)	294 (34.3)	132 (41.4)	
Male	749 (63.7)	562 (65.7)	187 (58.6)	
Race (%)				0.199
White	754 (64.2)	566 (66.1)	188 (58.9)	
Black	77 (6.6)	53 (6.2)	24 (7.5)	
Asian	29 (2.5)	20 (2.3)	9 (2.8)	
Hispanic	29 (2.5)	22 (2.6)	7 (2.2)	
Other	286 (24.3)	195 (22.8)	91 (28.5)	
BMI (kg/m2)	27.92 (24.47, 32.45)	28.37 (24.81, 32.67)	27.09 (23.54, 32.26)	**0.006**
CCI, [M(IQR)]	7.00 (5.00, 9.00)	7.00 (5.00, 8.00)	8.00 (6.00, 9.00)	**< 0.001**
Comorbidities, n (%)				
Cardiovascular disease	753 (64.1)	540 (63.1)	213 (66.8)	0.270
Chronic pulmonary disease	362 (30.8)	251 (29.3)	111 (34.8)	0.083
Liver disease	139 (11.8)	81 (9.5)	58 (18.2)	**< 0.001**
Renal disease	356 (30.3)	253 (29.6)	103 (32.3)	0.404
Diabetes	469 (39.9)	345 (40.3)	124 (38.9)	0.705
Malignancy	90 (7.7)	52 (6.1)	38 (11.9)	**0.001**
Dementia	61 (5.2)	38 (4.4)	23 (7.2)	0.079
AIDS	3 (0.3)	1 (0.1)	2 (0.6)	0.373
Infection site, n (%)				
Lung infection	278 (23.7)	193 (22.5)	85 (26.6)	0.164
Gastrointestinal infection	60 (5.1)	41 (4.8)	19 (6.0)	0.510
Genito urinary infection	209 (17.8)	161 (18.8)	48 (15.0)	0.157
Other	503 (42.8)	321 (37.5)	182 (57.1)	**< 0.001**
Intervention, n (%)				
Severity of illness, [M(IQR)]				
mSOFA	4.00 (2.00, 6.00)	4.00 (2.00, 6.00)	6.00 (4.00, 8.00)	**< 0.001**
Inflammatory biomarkers, [M(IQR)]				
NLR	8.75 (4.93, 15.78)	7.83 (4.61, 13.73)	11.68 (6.57, 21.04)	**< 0.001**
MLR	0.48 (0.25, 0.87)	0.44 (0.23, 0.78)	0.62 (0.33, 1.13)	**< 0.001**
PLR	4.83 (1.37, 139.80)	3.52 (1.18, 113.27)	54.16 (1.94, 214.64)	**< 0.001**

Abbreviations: BMI: body mass index; CCI: Charlson Comorbidity Index; AIDS: Acquired Immunodeficiency Syndrome; SOFA: Sequential Organ Failure Assessment; mSOFA: modified Sequential Organ Failure Assessment; NLR; Neutrophil-to-Lymphocyte ratio; MLR: Monocyte-to-Lymphocyte ratio, PLR: Platelet-to-Lymphocyte ratio. Bold text indicates *P* < 0.05

### Mortality in groups with different levels of inflammatory biomarkers

Table 2 compares mortality in groups with different levels of inflammatory biomarkers. Compared with the low levels of those inflammatory biomarkers (Q1), the high levels of inflammatory biomarkers group (Q3) of NLR, MLR, and PLR had higher mortality at 7-day, 14-day, 21-day and 28-day (*P* < 0.05).

**Table 2:** Mortality in groups with different levels of inflammatory biomarkers

		NLR					MLR					PLR			
	Total	Q1 < 5.86	5.86 ≤ Q2 < 12.58	Q3 ≥ 12.58	*P*		Q1 < 0.32	0.32 ≤ Q2 < 0.70	Q3 ≥ 0.70	*P*		Q1 < 1.90	1.90 ≤ Q2 < 90.21	Q3 ≥ 90.21	*P*
Mortality, n (%)															
7-day	186 (15.8)	42 (10.7)	52 (13.3)	92 (23.5)	**< 0.001**		50 (12.7)	47 (12.1)	89 (22.7)	**< 0.001**		46 (11.8)	57 (14.5)	83 (21.2)	**0.001**
14-day	259 (22.0)	57 (14.5)	78 (19.9)	124 (31.6)	**< 0.001**		66 (16.8)	73 (18.7)	120 (30.6)	**< 0.001**		62 (15.9)	84 (21.4)	113 (28.8)	**< 0.001**
21-day	296 (25.2)	68 (17.3)	93 (23.8)	135 (34.4)	**< 0.001**		78 (19.8)	85 (21.8)	133 (33.9)	**< 0.001**		75 (19.2)	97 (24.7)	124 (31.6)	**< 0.001**
28-day	319 (27.1)	70 (17.9)	100 (25.6)	149 (38.0)	**< 0.001**		79 (20.1)	98 (25.1)	142 (36.2)	**< 0.001**		77 (19.7)	105 (26.8)	137 (34.9)	**< 0.001**
ICU	233 (19.8)	50 (12.8)	69 (17.6)	114 (29.1)	**< 0.001**		54 (13.7)	68 (17.4)	111 (28.3)	**< 0.001**		56 (14.3)	70 (17.9)	107 (27.3)	**< 0.001**
Hospital	271 (23.1)	60 (15.3)	83 (21.2)	128 (32.7)	**< 0.001**		70 (17.8)	73 (18.7)	128 (32.7)	**< 0.001**		68 (17.4)	83 (21.2)	120 (30.6)	**< 0.001**

Data are presented as n (%), NLR, Neutrophil-to-Lymphocyte ratio; MLR, Monocyte-to-Lymphocyte ratio, PLR, Platelet-to-Lymphocyte ratio. Q1, low levels of inflammatory biomarkers; Q2, Medium levels of inflammatory biomarkers; Q3, high levels of inflammatory biomarkers. Bold values represent significant *p* values

### Associations of inflammatory biomarkers with 28-day mortality in septic patients with CAD

**Fig. 2:**
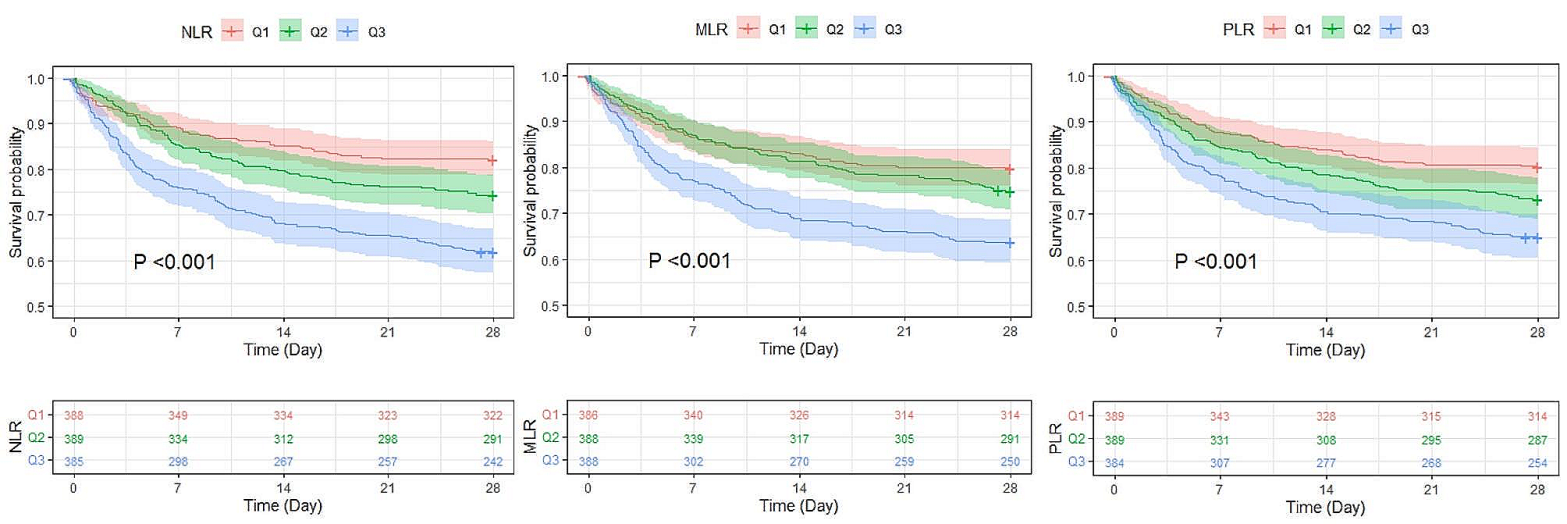
Kaplan‒Meier analysis analysis of the difference among different levels of inflammatory biomarkers

Kaplan‒Meier analysis showed that among the three inflammatory biomarkers (NLR, MLR and PLR), the group with high inflammatory biomarkers (Q3) had a higher 28-day all-cause mortality than the group with low inflammatory biomarkers (Q1) (*P* < 0.001) (Fig. 2). Univariate Cox proportional-hazard regression analysis showed that continuous variable NLR, PLR, and MLR were associated with 28-day mortality in septic patients with CAD. Among the three inflammatory biomarkers (NLR, MLR and PLR), the group with high inflammatory biomarkers (Q3) had a higher 28-day mortality than the group with low inflammatory biomarkers (Q1). Adjust for age, gender, BMI, race, cardiovascular disease, chronic pulmonary disease, liver disease, malignancy, dementia and mechanical ventilation, 28-day all-cause mortality of septic patients with CAD was significantly related to rising NLR levels (adjusted hazard ratio [aHR]: 1.02; 95% confidence interval [CI]: 1.01–1.02; *P* < 0.001), MLR levels (aHR: 1.29; 95%CI: 1.18–1.41; *P* < 0.001), and PLR levels (aHR: 1.0007; 95%CI: 1.0004–1.0011; *P* < 0.001). Meanwhile, the higher levels (Q3) group of NLR, MLR, and PLR also had a higher risk of 28-day all-cause mortality than the lower (Q1) group (Table 3).

**Table 3:** Cox proportional-hazards regression analysis for associations of inflammatory biomarkers with 28-day all-cause mortality in septic patients with CAD

	Unadjusted			Model 1			Model 2	
Variable	Hazard ratio (95% CI)	*P*		Hazard ratio (95% CI)	*P*		Hazard ratio (95% CI)	*P*
NLR	1.02(1.01,1.02)	**< 0.001**		1.02(1.01,1.02)	**< 0.001**		1.02(1.01,1.02)	**< 0.001**
Q1	ref	ref		ref	ref		ref	ref
Q2	1.47(1.08,1.99)	**0.014**		1.43(1.05,1.95)	**0.023**		1.42(1.04,1.93)	**0.027**
Q3	2.41(1.82,3.21)	**< 0.001**		2.31(1.73,3.07)	**< 0.001**		2.15(1.61,2.87)	**< 0.001**
MLR	1.30(1.19,1.41)	**< 0.001**		1.32(1.21,1.45)	**< 0.001**		1.29(1.18,1.41)	**< 0.001**
Q1	ref	ref		ref	ref		ref	ref
Q2	1.25(0.93,1.68)	0.141		1.15(0.85,1.55)	0.372		1.09(0.81,1.48)	0.557
Q3	1.98(1.51,2.61)	**< 0.001**		1.82(1.38,2.41)	**< 0.001**		1.69(1.27,2.24)	**< 0.001**
PLR	1.0008(1.0004,1.0011)	**< 0.001**		1.0007(1.0004,1.0011)	**< 0.001**		1.0007(1.0004,1.0011)	**< 0.001**
Q1	ref	ref		ref	ref		ref	ref
Q2	1.41(1.05,1.89)	**0.022**		1.34(1.00,1.81)	0.052		1.33(0.99,1.79)	0.060
Q3	1.96(1.48,2.59)	**< 0.0001**		1.83(1.38,2.43)	**< 0.001**		1.83(1.38,2.44)	**< 0.001**

HR, Hazard Ratio; CI, confidence interval; NLR, Neutrophil-to-Lymphocyte ratio; MLR, Monocyte-to-Lymphocyte ratio, PLR,

Platelet-to-Lymphocyte ratio. BMI, body mass index

Q1: low levels of inflammatory biomarkers;

Q2: Medium levels of inflammatory biomarkers;

Q3: high levels of inflammatory biomarkers

Model 1: adjust for age, gender, BMI, race

Model 2: adjust for age, gender, BMI, race, cardiovascular disease, chronic pulmonary disease, liver disease, malignancy, dementia, mechanical ventilation

Bold values represent significant *p* values

### Predictive value of 28-day all-cause mortality in septic patients with CAD

**Fig. 3:**
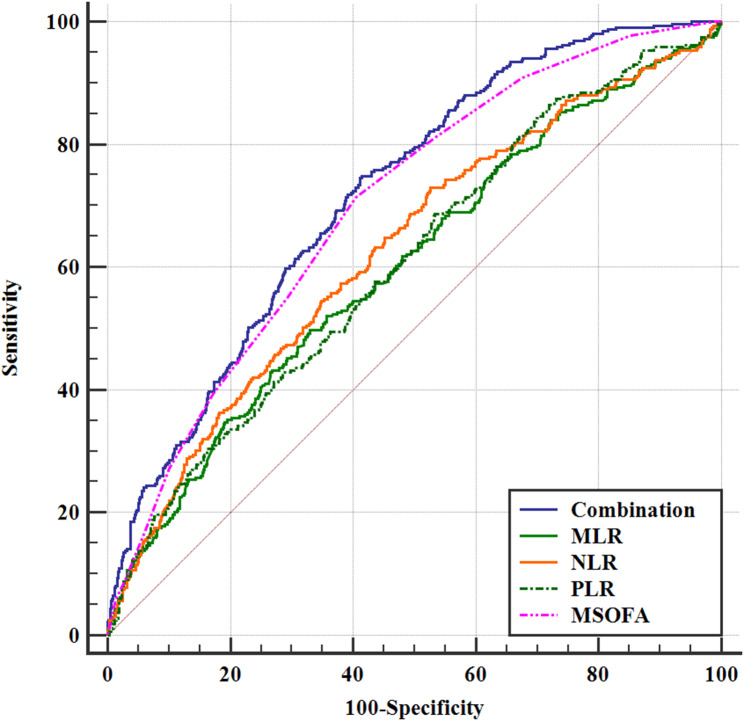
Prediction value of 28-day mortality in septic patients with CAD NLR: Neutrophil-to-Lymphocyte ratio; MLR: Monocyte-to-Lymphocyte ratio, PLR; Platelet-to-Lymphocyte ratio; mSOFA; modified sequential organ failure assessment

ROC analysis showed that the AUC of mSOFA scores, MLR, NLR, and PLR in predicting the 28-day mortality in septic patients with CAD was 0.699 (95%CI 0.672–0.725), 0.601 (95%CI 0.573–0.630), 0.627 (95%CI 0.599–0.655), and 0.606 (95%CI 0.577–0.634), respectively. Then the area under the curve (AUC) of the combined four indicators (mSOFA, NLR, MLR, PLR) in predicting the 28-day all-cause mortality in septic patients with CAD was 0.719 (95%CI 0.692–0.745). Thus, the combined index significantly improved the ability of predicting 28-day all-cause mortality compared to the predictive values of alone indicators (*P* < 0.05) (Table 4; Fig. 3).

**Table 4:** ROC analysis for 28-day all-cause mortality in septic patients with CAD

Variable	AUC	SE	95% CI	Cut-off value	Sensitivity	Specificity	Youden index J
Combination (MLR + NLR + PLR + MSOFA)	0.719	0.0158	0.692 to 0.745	> 0.233	74.92	58.53	0.335
MLR	0.601	0.0189	0.573 to 0.630	> 0.643	49.84	66.94	0.168
NLR	0.627	0.0186	0.599 to 0.655	> 7.301	73.04	47.31	0.204
PLR	0.606	0.0185	0.577 to 0.634	> 2.785	68.65	46.61	0.153
MSOFA	0.699	0.0163	0.672 to 0.725	> 4	71.47	59.46	0.309

*Abbreviations* NLR, Neutrophil-to-Lymphocyte ratio. MLR, Monocyte-to-Lymphocyte ratio. PLR, Platelet-to-Lymphocyte ratio. mSOFA, modified Sequential Organ Failure Assessment. AUC, Area Under the Curve. CI, Confidence Interval. SE, Standard Error

## Discussion

Our study demonstrated that high levels of inflammatory biomarkers (NLR, MLR, and PLR) were associated with 28-day all-cause mortality in septic patients with CAD. In addition, we found that using a combination of mSOFA scores with inflammatory biomarkers levels significantly improved the ability in predicting the 28-day all-cause mortality in septic patients with CAD. Our findings had important clinical significance because the ability to predict the 28-day risk of death in septic patients with CAD using easily accessible inflammatory biomarkers and a simple scoring system will allow clinicians to improve the diagnosis and treatment times of these patients.

Although neutrophil, monocyte and lymphocyte play a crucial role in the body’s defense against pathogens, a patient’s white blood cell count may present false negatives or may be delayed in reflecting the progression of inflammatory disease. Recent studies have demonstrated that white blood cell count ratios such as NLR, MLR, and PLR may act as potential inflammation biomarkers in a variety of diseases. For example, Shi et al. found that NLR levels on day 5 in hospitalised patients were independently associated with mortality in septic patients [33], while Shen et al. reported that a high level of PLR on admission was associated with mortality in septic patients [34]. Furthermore, NLR has been shown to independently predict cardiac mortality in patients with stable CAD; a dose-response model of NLR demonstrated that the risk of death increased when the NLR was higher than 2.42 [35]. Chen et al. found that a high level of NLR was associated with myocardial dysfunction [36]. Similarly, Bressi et al. showed that elevated NLR, but not PLR, before surgery was an independent predictor of major adverse cardiac events in CAD patients [37]. Our study further extended the role of inflammatory biomarkers to septic patients with CAD. We found that non-survival group had higher NLR, MLR, and PLR values compared to the survival group, and that high NLR and PLR levels were independent risk factors for 28-day mortality in septic patients with CAD. Our findings suggested that inflammatory biomarkers were also associated with poor prognosis in the disease comorbidities, and that changes in inflammatory biomarkers reflected septic inflammation and progression of myocardial damage.

We further explored the ability of inflammatory biomarkers in predicting 28-day all-casue mortality in septic patients with CAD. We found that NLR, MLR, and PLR predicted 28-day mortality in septic patients with CAD. The AUCs of NLR, MLR, and PLR were 0.627, 0.601, and 0.606, respectively. Similarly, Liu et al. reported that the AUC of predicting 28-day mortality in septic patients by NLR was 0.634, and its sensitivity and specificity were 78.3% and 50%, respectively [38]. Shi et al. found that the AUC of predicting mortality in septic patients using NLR on day 5 of admission was 0.589, and the sensitivity and specificity were 32.3% and 83.3% [33], respectively. Together, these findings suggested that the inflammatory biomarkers were similar in predicting mortality. In addition, these studies also suggested that single inflammatory biomarkers were insufficient to predict disease mortality accurately. Thus, in order to improve the predictive value of mortality in such patients, a better combination of indicators may be required.

SOFA scores were an important part of sepsis-3 criteria [27]. Previous studies have reported that SOFA scores were a useful early prognostic marker for predicting 28-day mortality in septic patients [39]. However, in recent years, there is a more critical attitude towards the SOFA score, as many studies have identified the limitations of the original version of the SOFA score in various accompanying diseases (COPD, chronic kidney disease, malignancies, etc.). At the same time, modifications of the SOFA score regarding the presence of individual organ system failure (mSOFA) have been developed in recent years. Due to the reduced importance of the original version of the SOFA score, especially in the case of cardio-vascular failure, and the unreliable criterion for CAD, the final results are also less reliable [24, 40]. Therefore, in the present study, we combined mSOFA scores rather than SOFA socre with inflammatory biomarkers values to predict 28-day all-cause mortality of septic patients with CAD. Our study found that although the ability of mSOFA scores in predicting the 28-day all-cause mortality of septic patients with CAD was insufficient, using a combination of inflammatory biomarkers with mSOFA scores could improve the ability of predicting 28-day mortality in patients with sepsis and CAD. These findings have important clinical significance, the diagnosis and treatment times of septic patients with CAD can be improved and 28-day all cause mortality of these patients can be predicted by using a combination of these easily accessible inflammatory biomarkers and a simple scoring system.

There were some limitations in this study. First, it was imperative to acknowledge that the MIMIC-IV database collects data over a time span of more than a decade. The ICD-9 did not have classification for defining sepsis, so previous studies identified septic patients in the MIMIC database by using codes. Due to changes in the definition of sepsis, patients identified as sepsis according to the sepsis 3.0 definition may include individuals who were not originally diagnosed with sepsis, potentially impacting treatment decisions. However, due to temporal drift in the MIMIC database, we were unable to extract septic patients based on different periods of sepsis definitions in the database. Our findings might be influenced by changes in the sepsis guidelines that occurred during this period, which could affect the practical relevance of our results. Secondly, CAD was defined according to the codes 410–411 (ICD-9) and I20-I21 (ICD-10) from disease history and medical record diagnosis, including previous myocardial infarction, or acute coronary syndrome etc. It was unclear whether these patients with CAD underwent coronary angiography and belong to obstructive coronary artery disease. Future prospective studies are needed to verify our conclusions. Thirdly, it is necessary to identify the source of the infection and the likely bacteria causing the sepsis, we cannot confirm the associations between different bacteria and different inflammatory profiles that affect NLR in particular due to no available additional data [41–43].

## Conclusions

Our study confirmed that high NLR, MLR, and PLR were associated with 28-day mortality in septic patients with CAD. Our results also demonstrated that using a combination of mSOFA scores and inflammatory biomarkers values significantly improve the diagnostic efficiency in predicting the 28-day mortality in septic patients with CAD. In the future, we need to combine laboratory tests, clinical symptoms, and scores to construct a simple, timely and efficient predictive model for the short-term risk of mortality in septic patients with CAD, in order to increase early intervention and decrease the risk of poor prognosis of these patients.

## Data Availability

The datasets generated and/or analyzed during the current study are available from the corresponding author on reasonable request.
